# Thyroid nodule with cytological outcome of indeterminate lesion with low risk of malignancy found to be parathyroid adenoma. A case report and minireview of literature

**DOI:** 10.3389/fendo.2025.1474440

**Published:** 2025-02-21

**Authors:** Letizia Meomartino, Mattia Rossi, Gloria Selvatico, Ruth Rossetto Giaccherino, Loredana Pagano

**Affiliations:** Endocrinology, Diabetology and Metabolism, Department of Medical Sciences, University of Turin, Turin, Italy

**Keywords:** intrathyroidal parathyroid adenoma, MIBI scan uptake, choline PET uptake, FNA, indeterminate cytology, thyroid nodule

## Abstract

Primary hyperparathyroidism (PHPT) is the third most common endocrine disorder, typically caused by a single parathyroid adenoma. The diagnosis of PHPT is biochemical, and the localization of abnormal parathyroid glands is usually achieved through a combination of ultrasound and technetium-99m sestamibi (99mTc-MIBI) scans. In some cases, newer imaging modalities, such as positron emission tomography-computed tomography (PET-CT) with 18F-fluorocholine or 11C-methionine, are used as second-line methods. Consequently, parathyroid tissue (PTT) is not typically sampled by fine needle aspiration biopsy (FNAb). However, with an incidence ranging from 9% to 22%, the affected parathyroid gland may present in an ectopic location, with the thyroid gland being a possible site. In intra-thyroidal parathyroid adenomas (IPAs), the differential diagnosis with thyroid nodules can be challenging due to similar ultrasound features and the potential uptake of 99mTc-MIBI by some thyroid nodules. As a result, such lesions may sometimes undergo unintentional cytological examination, leading to the risk of misinterpretation as cytologically indeterminate thyroid lesions. This can result in both misdiagnosis and inappropriate surgical approach. For this reason, a routine evaluation of calcium-phosphorus metabolism could prove beneficial as part of the diagnostic workup for cytologically indeterminate thyroid nodules, especially when surgery is planned. To support this diagnostic approach, we present a mini-review of the literature on this topic, along with a case report of an IPA misinterpreted as an indeterminate thyroid lesion (TIR3A, according to the Italian Society for Anatomic Pathology and Cytology–Italian Thyroid Association 2014 classification system), diagnosed following the preoperative incidental detection of hypercalcemic PHPT.

## 1 Introduction

Primary hyperparathyroidism (PHPT) is the third most common endocrine disorder, caused by excess parathyroid hormone (PTH) production, leading to elevated serum calcium levels (1, 2). The most common etiology is a solitary parathyroid adenoma (up to 85% of cases), followed by primary parathyroid hyperplasia (10-15%), and parathyroid carcinoma (less than 1%) (1, 3–5).

Parathyroid tissue (PTT) is not routinely sampled by fine-needle aspiration biopsy (FNAb), as the diagnosis of PHPT is biochemical and thyroid ultrasound in association with technetium-99m sestamibi (99mTc-MIBI) scans is generally sufficient to detect abnormal parathyroid glands (6). Newer nuclear medicine imaging modalities, such as positron emission tomography-computed tomography (PET-CT) with 18F-fluorocholine, 11C-methionine, or 11C-choline, have proven useful as second-line methods (1, 7).

Since the preferred treatment is minimally invasive parathyroidectomy, accurate preoperative localization of hyperfunctioning parathyroid glands is crucial (3, 8).

However, intra-thyroidal parathyroid adenomas (IPAs) can be difficult to differentiate from thyroid nodules due to overlapping ultrasound features and the potential uptake of 99mTc-MIBI by certain thyroid nodules (1, 9). Since the evaluation of the calcium-phosphorus metabolism is not routinely performed prior FNAb and the fact that 99mTc-MIBI uptake is a known risk modifier for thyroid malignancy (10), such lesions may sometimes undergo unintentional cytological examination. Moreover, FNAB has shown limited diagnostic performance in these cases due to the cytomorphological overlap with thyroid lesions, such as follicular neoplasms, making differential diagnosis even more complex (6, 11).

Consequently, the literature reports several cases of cytologically indeterminate thyroid lesions later confirmed as parathyroid adenomas (9, 11–16).

Hereby, we present a case report of a cytologically diagnosed low-risk indeterminate thyroid lesion (TIR3A, according to the Italian Society for Anatomic Pathology and Cytology–Italian Thyroid Association 2014 classification system) (17), which was later confirmed as PTT upon histological examination, along with a mini-review of the literature on this topic.

## 2 Case report

A 46-year-old woman with multinodular non-toxic goiter (TSH 2.735 mIU/L, fT4 9 ng/L) presented with a 50 mm solid, well-defined, isoechoic nodule in the left thyroid lobe with perinodular and endonodular vascularization. The nodule, initially identified as a thyroid lesion at another institution, underwent FNAb in the same institution, with cytological diagnosis of low-risk-indeterminate thyroid lesions [TIR3A according to the 2014 Italian Society for Anatomic Pathology and Cytology–Italian Thyroid Association classification system (17)]. Immunohistochemistry revealed mild positivity for chromogranin A.

Due to the nodule’s size, the patient was referred for surgical treatment. Preoperative assessments included serum calcitonin (negative) and evaluation of calcium-phosphate metabolism, which revealed findings consistent with primary hypercalcemic hyperparathyroidism (**Table 1**). Her medical history was negative for renal stones or kidney disease that could suggest secondary hyperparathyroidism, and there was no relevant family history.

**Table 1:** Preoperative biochemical blood test and evaluation of complications of hyperparathyroidism.

	Results	Normal range
Calcium (mmol/L)	2.8	2.2-2.65
Albumin (g/L)	42	35-52
Ionized Calcium (mmol/L)	1.57	1.12-1.32
PTH (ng/L)	488	6.5-36.4
25-OH-Vitamin D (mcg/L)	31	>30
Creatinine (mg/dl)	0.78	0.72-1.18
eGFR (CKD-EPI) (ml/min)	73	≥90
	Results	
Three-site DXA	- Distal 1/3 radius: Z-score -2.9 and T-score – 3.5; - Femoral neck: Z-score -0.3 and T-score -0.9, Femoral total Z-score -0.7 and T-score -1.0; - Lumbar spine (L1-L4): Z-score -0.5 and T-score -1.1.	
Abdomen US	- Presence of nephrolithiasis.	

PTH, Parathyroid hormone; DXA, Dual Energy X-ray Absorptiometry; US, ultrasound; eGFR, Estimated glomerular filtration rate; CKD-EPI, Chronic Kidney Disease Epidemiology Collaboration.

A 99mTc-MIBI scan showed extensive and uneven uptake on delayed images, corresponding to the large left thyroid nodule. Additionally, a Dual Energy X-ray Absorptiometry (DXA) scan and abdominal ultrasound confirmed the presence of complications, including osteoporosis and nephrolithiasis (**Table 1**).

In July 2023, the patient underwent left loboisthmectomy due to the absence of a cleavage plane between the nodule and the thyroid gland. Intraoperative PTH levels decreased rapidly, from 310 ng/L to 10 ng/L after 20 minutes, representing a reduction of over 90%. Histological examination revealed a “thyroid gland within a well-differentiated neuroendocrine neoplasm with initial focal capsular penetration and a low proliferative index (probable parathyroid adenoma),” with a Ki-67 index of 2%, and no suggestive features of atypical adenoma or carcinoma.

The patient recovered well postoperatively and was discharged on the 4th day without complications. Serum calcium and PTH levels normalized rapidly post-surgery (PTH 20.4 ng/L, serum calcium 2.13 mmol/L, albumin 35 g/L, albumin-corrected calcium 2.2 mmol/L on the first day). Four months postoperatively, mild hyperparathyroidism persisted (PTH 63 ng/L), which was considered secondary to hypocalcemia and vitamin D deficiency (calcium 2.17 mmol/L, albumin 41 g/L, 25-OH-vitamin D 14 mcg/L). Six months after surgery, following optimization of calcium and vitamin D levels, the calcium-phosphate profile had completely normalized (calcium 2.2 mmol/L, albumin 41 g/L, phosphorus 0.8 mmol/L, PTH 32 ng/L, 25-OH-vitamin D 28 mcg/L).

## 3 Minireview of literature

Ectopic parathyroid glands result from aberrant migration during the early stages of development. In patients with PHPT, the incidence of ectopic adenomas ranges from 9% to 22% (1).

IPAs are a rare cause of PHPT, with an incidence ranging from 0.7% to 6%, and more commonly (> 85%) appear to involve the lower parathyroid gland (1, 18). The wide range of reported incidences may be attributed to the limited data in the literature, which is predominantly based on retrospective studies, case reports, and few reviews (1, 19–29).

In particular, **Table 2** summarizes all the studies and case reports we were able to identify that describe IPAs initially cytologically misinterpreted as indeterminate thyroid lesions.

**Table 2:** Cases of parathyroid tissue with a cytological diagnosis of indeterminate thyroid lesions, eventually confirmed parathyroid adenomas.

	Article types	FNAb patients (N)	Undeterminate Nodules (N)	Confirmed IPAs	History of PHPT	FNAb result	Diagnosis	Surgery	Histologically confirmed
Rossi et al, 2003 (12)	CR	1	1	1	–	Thyroid follicular lesion	Histology	1	1
Bo et al, 2017 (9)	RS	4765	4765	20	8	Bethesda III (N: 9); Bethesda IV (N: 6).	ThyroSeq v2; biochemical PHPT	9	8
Cho et al, 2017 (11)	RS	34	5	5	–	Bethesda III (N: 3); Bethesda IV (N: 2).	PTH-ICC; GATA3-ICC; ThyroSeq v2; 99mTc-MIBI scan	–	–
Domingo et al, 2017 (13)	RS	60	60	13	16	Bethesda III (N: 6); Bethesda IV (N: 7)	Cytology, Afirma GEC; PTH-ICC	–	–
Yabuta et al, 2011 (14)	RS	2	1	1	0	Follicular tumor	Biochemical PHPT; PTH washout	2	2
Paker et al, 2010 (15)	CR	1	1	1	1	Hürthle cell thyroid neoplasm	Biochemical PHPT; 99mTc-MIBI scan	1	1
Odashiro et al, 2006 (16)	CR	1	1	1	1	Thyroid microfollicular neoplasm	Histology	1	1

CR, case report; RS, retrospective study; FNAb, fine needle ago biopsy; IPAs, intrathyroidal adenomas; N, number; PHPT, primary hyperparathyroidism; FLUS, follicular lesion of undetermined significance; PTH, parathyroid hormone; ICC, immunocytochemistry; GEC, Gene Expression Classifier; 99mTc-MIBI, technetium-99m sestamibi.

### 3.1 Diagnosis/localization

In HPTH, an accurate preoperative localization is essential to assess the extent of the surgical procedure as MIP is currently the first choice when feasible (30, 31). While thyroid ultrasound and 99mTc-MIBI scans are generally sufficient to detect abnormal parathyroid glands (6), IPAs are often difficult to identify due to their similar ultrasound characteristics to thyroid nodules and the potential uptake of 99mTc-MIBI by certain thyroid nodules (1, 9, 32). Correct diagnosis is crucial, as it may influence the surgical approach, shifting from a total thyroidectomy to a lobectomy in some cases. However, as seen in the cases listed in **Table 2**, the proper diagnosis is generally achieved using a combination of different diagnostic techniques, rather than relying on a single parameter.

#### 3.1.1 Ultrasound

As mentioned previously, ultrasound is usually the first tool for localizing parathyroid lesions, as it is cost-effective, noninvasive, widely available, and radiation-free (1, 3, 33). However, its sensitivity is highly variable, ranging from 57% to 84% (34, 35), with a nadir of 47% when thyroid goiter coexists (35).

When located in typical sites, enlarged parathyroids glands are usually easily recognized as homogenous hypoechoic solid masses, with oval or oblong shape and peripheral vascularity, often with a typical extrathyroidal feeding vessel (8, 11, 36, 37). However, ultrasound is well-known to be an operator-dependent technique, and when lesions are located within the thyroid capsule, these features may be indistinguishable from those of thyroid nodules, making the differential diagnosis between IPAs and thyroid nodules challenging, or even impossible, sonographically (1, 3, 11, 33).

As a result, the sensitivity of ultrasonography for detecting IPAs is significantly reduced with a variable range from 29% to 67% (1, 5, 29, 32, 38, 39).

Yabuta et al. found that the most common ultrasound features of IPAs included a hypoechoic solid mass with smooth borders, a regular tumor shape and a characteristic hyperechoic line on the ventral surface of the adenoma, produced by the thin capsule between the parathyroid gland and the thyroid parenchyma (14).

In a recent study, Haciyanli et al. (21) proposed several criteria to distinguish IPAs from thyroid nodules:

- Absence of PTT in its usual anatomical location;
- Hypervascularity and the presence of an intraglandular polar vessel;
- A more homogeneous hypoechoic pattern than the thyroid nodules;
- Absence of halo sign;
- Well circumscribed lesion.

Nevertheless, the differential diagnosis may be troublesome. Since IPAs usually mimic thyroid nodules with intermediate-high risk ultrasound characteristics (40), this can lead to unnecessary FNAb and potentially to misinterpretation of cytological results.

#### 3.1.2 Nuclear imaging

The main localization method for parathyroid lesions is 99mTc-MIBI scan, with a sensitivity of up to 88%, when studying a single-gland disease (41). However, when detecting IPAs, some studies report significantly lower sensitivity, around 60% (1).

This technique does have certain limitation, such as an unsatisfactory specificity, the ability of some thyroid nodules to uptake 99mTc-MIBI (6, 11) and poor spatial resolution (33).

Therefore, functional imaging cannot reliably distinguish between a thyroid tissue and a hyperplastic parathyroid gland, as normal thyroid gland or thyroid nodule also uptake this radioisotope (14). The most common cause of false positives is solid thyroid nodules (42), including toxic thyroid adenomas and oncocytic tumors (3). Additionally, delayed tracer washout has been observed in thyroid carcinoma (42), and 99mTc-MIBI uptake may strengthen the indication for FNAb, alongside factors such as nodule size and ultrasound risk level (10).

On the other hand, false negatives can occur in the presence of smaller adenomas or concomitant thyroid multiglandular disease (1). Therefore, a negative 99mTc-MIBI scan, especially in patients with a history of hyperparathyroidism, should not exclude the diagnosis. When a false negative is suspected, newer nuclear imaging modalities such as PET-TC with ^18^F-fluorocholine, ^11^C‐methionine or ^11^C-choline, have proven useful as second-line methods (1, 7).

^11^C-methionine is retained in the parathyroid adenomas due to its involvement in the synthesis of parathyroid hormone precursors (43). The reported sensitivity of ^11^C-methionine PET/CT is 72–86% (44), but a recent retrospective study (43) demonstrated high sensitivity (98%) and specificity (93%) for ^11^C-methionine PET/CT. This technique appears to be particularly useful in cases of suspected ectopic parathyroid adenoma, prior unsuccessful surgery or inconclusive previous imaging (7).

In recent years, both ^11^C-choline and ^18^F-fluorocholine PET/CT have gained attention as functional imaging techniques for parathyroid lesions (7).

The pooled sensitivity of ^11^C-choline PET/CT is reported at 86% (34), although Noltes et al. observed a higher sensitivity of 97% in patients with primary hyperparathyroidism who underwent surgery following non-conclusive first-line imaging (44).

A major limitation of both ^11^C-methionine and ^11^C-choline imaging is the complex and costly production process of tracers, necessitating an on-site cyclotron due to their short half-lives (7, 45).

In contrast, ^18^F-fluorocholine PET/CT offers high sensitivity (90–96%) (44) and has the advantage of a longer half-life compared to ^11^C-choline PET/CT and ^11^C-methionine PET/CT, removing the need for on-site production (7, 43, 44). Additionally, thanks to a more amenable positron range, ^18^F-fluorocholine PET/CT offers higher spatial resolution than 99mTc-MIBI scan and ^11^C-methionine PET/CT, enabling the detection of smaller or ectopic parathyroid adenomas, including IPAs (1).

False-negative findings with choline PET may occur in smaller adenomas or in adenomas with a low number of oxyphilic cells, and IPAs due to the masking effect of thyroid uptake (45). False-positive findings are often due to thyroid nodules that also may take up the radiotracer (45). It has been suggested that ^18^F-fluorocholine PET/CT may be particularly useful in the study of thyroid nodules with indeterminate cytology to estimating the risk of malignancy, due to the high negative predictive value (96%) (46). Thus, the presence of a ^18^F-fluorocholine-avid thyroid nodule could favor the decision to perform FNAb.

Recent guidelines do not specifically address the potential presence of IPAs (47). As noted in recent studies, ^18^F-fluorocholine-PET/CT has shown high accuracy in detecting benign parathyroid lesions, particularly when other imaging techniques are negative or discordant (48). However, data on IPAs remain limited.

Therefore, given the limited available data and the limitations of the various techniques discussed above, no single imaging modality is currently considered superior for differential diagnosis.

#### 3.1.3 Fine needle aspiration biopsy and ancillary techniques

For all the reasons outlined above, in case of IPAs, inadvertent sampling of PTT by FNAb is a common pitfall in the diagnostic process of IPAs, particularly when a patient’s history of PHPT is not provided (49).

The prevalence of inadvertently sampling unsuspected PTT, presenting as thyroid lesions, has been reported to be up to 0.4% (6, 9, 11). Although cytomorphology alone may sometimes allow for a correct diagnosis, the ability of routine FNAb to distinguish between thyroid tissue and PTT is limited due to cytomorphological overlap, especially when parathyroid adenomas are located within the thyroid gland. In these cases, the sensitivity rages from 40% to 86% (6, 11, 37, 49).

Parathyroid cells share similar cytomorphological features with thyroid follicular cells. Consequently, PTT in FNAb sample can easily be confused with papillary thyroid carcinoma, Hürthle cell thyroid neoplasms and lymphocytic thyroiditis (19, 50). This is due to the presence of papillary fragments, epithelial cells arranged in microfollicular pattern, colloid-like material, anisokaryosis and the presence of oxyphil cells and naked nuclei of chief cells (50, 51), all of which may mimic neoplastic thyroid lesions (51).

Three different types of parathyroid cells have been described (52):

- Chief cells, which are similar to follicular cells of the thyroid.
- Oxyphil cells, which resemble Hürthle cells of the thyroid.
- Water clear cells, characterized by clear cytoplasm.

In these cases, a thorough medical history is essential. Known history of PHPT in the presence of a suspicious lesion justifies the request for specific ancillary tests that have been shown to be useful in distinguishing between parathyroid and thyroid lesions.

However, as in our case, the patient may be asymptomatic or may present with inconsistent symptoms of PHTP, which can lead to diagnostic delays (1). Moreover, calcium-phosphorus metabolism tests are not routinely required as part of the preliminary evaluations prior to FNAb.

Indeed, several cases of IPAs with cytological diagnosis of thyroid lesions have been reported in the literature, where the use of ancillary techniques allowed for the correct diagnosis on cytology specimens (9, 11, 13–15).

Therefore, in cases of high clinical suspicion for HPTH (e.g. a known history of HPTH), in the presence of a cytologically indeterminate intrathyroidal lesion and inconclusive imaging, the literature suggests that the use of ancillary techniques on FNAb specimens may be useful, as described below.

Firstly, the integration of cytological analysis with liquid-based preparation, in addition to the conventional smear, appears to be useful in the differential diagnosis (49). Common cytological features of parathyroid cells observed in liquid-based preparation include cells with small, dark nuclei, moderate to scant lacy cytoplasm, and, in some cases, oncocytic differentiation, clustered into small groups (49).

Park et al. compared the results of conventional smear and liquid-based preparation using ThinPrep and SurePath methods. Common features of parathyroid lesions observed in both conventional smear and liquid-based preparation included microfollicular structure, small round-to-oval nuclei with lymphocyte-like chromatin, and naked nuclei in the background. Specimens prepared using both ThinPrep and SurePath methods showed higher nuclear detail and better defined cytoplasm (53).

Moreover, bubbly or vacuolated cytoplasm was only observed in liquid-based preparation. Other findings included oxyphilic parathyroid cells, which may resemble Hürthle cells of the thyroid gland, although Hürthle cells tend to have larger sizes and plumper cytoplasm than oxyphilic parathyroid cells, and white blood cells in the background, which can be useful for differential diagnosis (53), as the nuclear size of parathyroid cells is similar to or smaller than that of inflammatory cells, while the nuclear size of follicular cells is larger (53).

However, the cytological features of parathyroid cells are various, and the liquid-based preparation may not be available at all centers.

Another additional method is the measurement of PTH in the washout fluid from FNAb, which has been shown to be helpful when hyperfunctioning parathyroid glands are suspected bat cannot be clearly identified via ultrasound examination, FNAb or 99Tc-MIBI scan (3, 13). Cansu et al. demonstrated that PTH-washout is superior to cytological examination for the localization of parathyroid adenomas in patients with negative imaging results (3). Furthermore, Abdelghani et al. showed that PTH-washout is highly sensitive and specific for the identification of PTT sampling in FNAb samples (54). In a recent retrospective study, PTH-washout reached 100% sensitivity and specificity (55). However, a key limitation of this technique is that a defined PTH-washout cut-off value for PTT has not yet been established. Various cut-off values have been proposed: Maser et al.’s suggestion that a PTH level above the normal reference range is diagnostic, while Abdelghani et al., and other specialists, proposed that PTH levels in the washout fluid should exceed serum levels, thus defining a washout to serum PTH ratio greater than 1 (3, 49). It has also been reported that a PTH washout value in FNAb specimens greater than 245 pg/mL strongly correlates with PTT (11). Another major limitation of this method is the labile nature of PTH, which requires specific preservation and transport conditions for the assay, limiting its widespread use (13).

Another ancillary study is the use of immunocytochemical staining on FNAb specimens, but this is only possible if cell block material is available. Staining for PTH and thyroglobulin has proven useful in distinguishing between PTT and thyroid tissue (13, 19, 49). Sardana et al. observed high specificity and sensitivity for PTH immunostain, with values of 100% and 85.7% respectively (6). False negatives can occur if there is insufficient hormone storage within individual cells (49). Therefore, thyroglobulin staining serves as a control to exclude the presence of thyroid tissue (49). Other immunocytochemical markers of neuroendocrine differentiation, such as chromogranin and synaptophysin, may also support the diagnosis (50, 56). The use of GATA-3 immunostaining has been proposed as a marker of parathyroid tissue, although it is less specific than PTH, as its positivity can be observed in other tumors as well as in lymphocytes (6). However, immunocytochemical staining is not routinely performed, and not all FNAb specimens are suitable for cell blocks preparation, as this technique requires a minimal level of cellularity (6). Some authors (6, 57, 58) have performed immunocytochemical staining on air-dried cytology smears or liquid-based cytology slides, but this approach is not yet considered standard practice.

Finally, emerging techniques, such as next-generation sequencing (NGS), enable the simultaneous identification of a broad spectrum of genetic alterations, requiring only minimal nucleic acid samples extracted from FNAb specimens. Molecular testing programs, such as the gene expression classifier (GEC) test (commercially known as Afirma, Veracyte, South San Francisco, Calif.) and Multi-Gene ThyroSeq Next-Generation, have been shown to be useful in confirming a parathyroid origin (13, 59, 60), but they remain costly and are not always available.

## 4 Conclusion

In conclusion, IPAs are a rare cause of PHPT and represent a diagnostic challenge, especially when the patient’s history of PHPT in unknown.

Currently, the available data are limited and primarily consists in case reports and small retrospective studies with heterogeneous populations, which complicates comparisons of findings. However, as illustrated by our case, when indeterminate or follicular neoplasm cytology is observed on FNAb of intrathyroidal lesions in patient with biochemical evidence of PHPT, IPAs should always be considered in the differential diagnosis of thyroid lesions.

For this reason, the routine evaluation of calcium-phosphorus metabolism could prove beneficial as part of the diagnostic workup of cytologically indeterminate thyroid nodules, especially when surgery is planned, mirroring the exclusion of medullary thyroid carcinoma through calcitonin screening in such patients. As a matter of fact, the identification of an IPA could significantly influence surgical extent and technical approach (10).
